# Current status of endoscopic ultrasound in the diagnosis of intraductal papillary mucinous neoplasms

**DOI:** 10.1002/deo2.413

**Published:** 2024-07-21

**Authors:** Eizaburo Ohno, Teiji Kuzuya, Naoto Kawabe, Kazunori Nakaoka, Hiroyuki Tanaka, Takuji Nakano, Kohei Funasaka, Ryoji Miyahara, Senju Hashimoto, Yoshiki Hirooka

**Affiliations:** ^1^ Department of Gastroenterology and Hepatology Fujita Health University School of Medicine Aichi Japan; ^2^ Department of Gastroenterology and Hepatology Fujita Health University Bantane Hospital Aichi Japan

**Keywords:** contrast‐enhanced harmonic EUS, EUS, EUS‐guided fine needle aspiration, intraductal papillary mucinous neoplasms, mural nodule

## Abstract

The new Kyoto guidelines for the management of intraductal papillary mucinous neoplasm (IPMN) provide evidence‐based recommendations for the diagnosis and treatment of IPMN. Endoscopic ultrasonography (EUS) is a diagnostic modality with a high spatial resolution that allows detailed observation and obtaining cyst fluid or tissue samples via EUS‐guided fine needle aspiration (EUS‐FNA). Currently, EUS is an indispensable examination method for the diagnosis of pancreatic diseases. On the other hand, there have been concerns that EUS imaging tends to be highly operator‐dependent, and may lack objectivity. Previous guidelines have assigned EUS as an option for patients with worrisome features. However, recent reports indicate that the sensitivity of EUS for the diagnosis of mural nodules (MNs) is more than 90%, comparable or superior to that of contrast‐enhanced computed tomography or magnetic resonance cholangiopancreatography. The specific advantages of EUS in the diagnosis of IPMN are: (1) high spatial resolution imaging for the diagnosis of MNs, (2) contrast‐enhanced EUS for differentiation of intra‐cystic MNs from mucous clots, and (3) pathological diagnosis using EUS‐FNA and differential diagnosis of a pancreatic cystic tumor by cystic fluid analysis. In order to utilize EUS in the diagnosis of IPMN, endoscopists are required to have the skills to provide sufficiently objective imaging findings.

## INTRODUCTION

Intraductal papillary mucinous neoplasm (IPMN) is a pancreatic epithelial neoplasm characterized by mucus production, associated cyst formation, and main duct dilation.^1^ Several guidelines have been reported to manage the treatment of IPMN and other pancreatic cystic lesions (PCLs), including international evidence‐based guidelines (Kyoto Guidelines 2023), guidelines by the European Study Group (2018), and American Gastroenterological Association (2015).^2^, ^3^, ^4^ IPMN is one of the most frequently diagnosed lesions among PCLs. IPMN is an epithelial neoplasm that slowly progresses from adenoma to adenocarcinoma, and the diagnosis of malignancy is presumed based on the morphologic features of IPMN: cyst size, main pancreatic duct (MPD) size, and nodules within the cyst. IPMN is reported to be a “high‐risk group for pancreatic cancer” because IPMN has the risk of malignant transformation (IPMN‐derived carcinoma) and the development of concomitant invasive pancreatic ductal carcinoma.^5^, ^6^, ^7^, ^8^ Endoscopic ultrasonography (EUS) has a high spatial resolution, which allows for a more detailed image than other diagnostic modalities.^9^ EUS‐guided fine needle aspiration (EUS‐FNA) was first reported by Vilmann in 1992 and has since been widely used in the pancreaticobiliary field, not only for tissue sampling for pathological diagnosis but also as an essential test for comprehensive genomic profile tests.^10^, ^11^

This work reviews the current role and the usefulness of EUS in the diagnosis of IPMN to date with respect to the following items.

1. The utility of EUS in distinguishing malignant from benign IPMN compared to other diagnostic modalities.
2. The advantages of contrast‐enhanced EUS/contrast‐enhanced harmonic EUS in IPMN diagnostics.
3. Pathological diagnosis using EUS‐FNA and differential diagnosis of pancreatic cystic tumor by cystic fluid analysis.

## THE ROLE OF EUS BASED ON THE FORMER INTERNATIONAL CONSENSUS GUIDELINES FOR IPMNs

**FIGURE 1:**
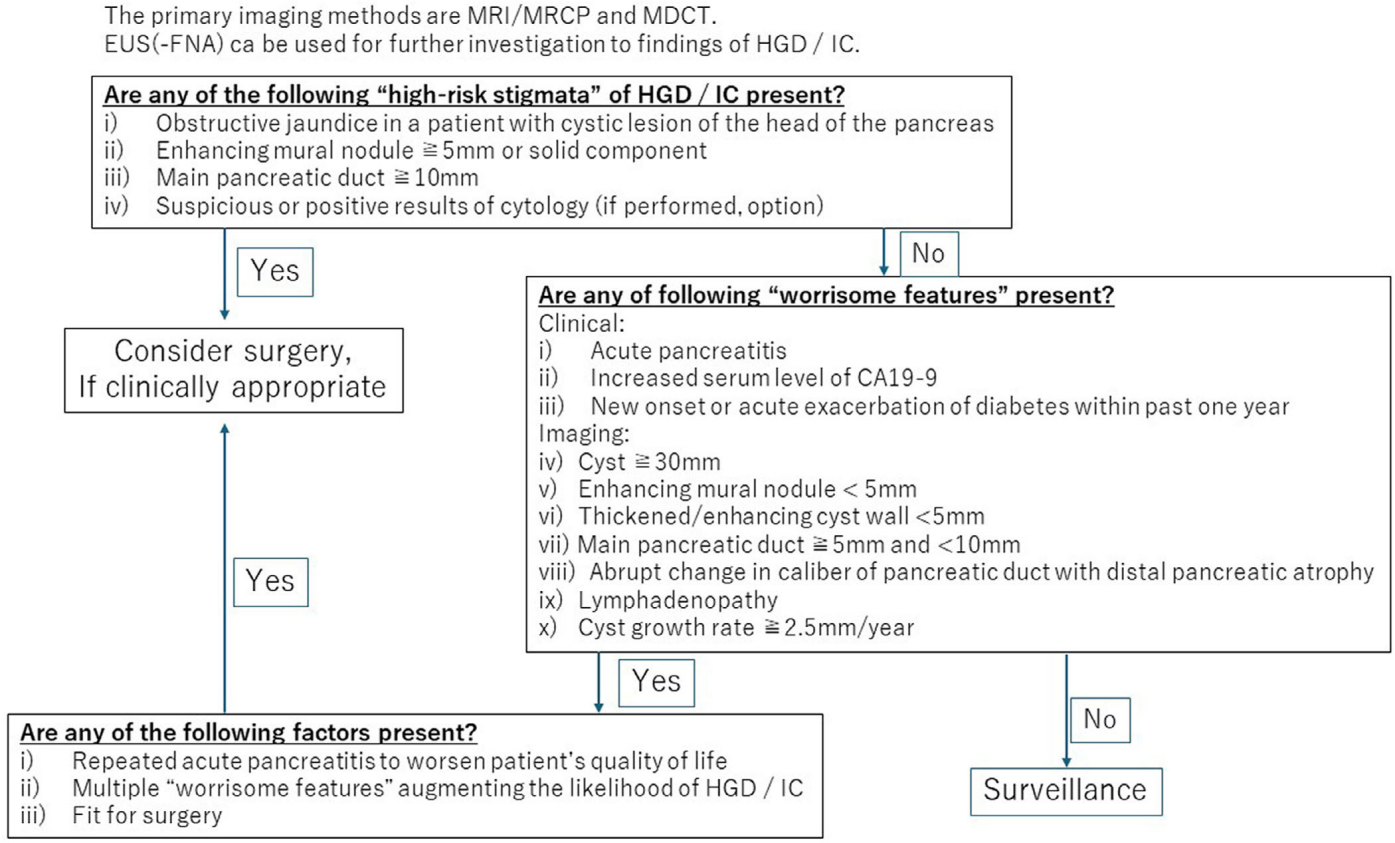
Diagnostic algorithm of intraductal papillary mucinous neoplasm based on Kyoto guidelines. HGD, high‐grade dysplasia; IC, invasive carcinoma; MDCT, multidetector computed tomography; MRCP, magnetic resonance cholangiopancreatography; MRI, magnetic resonance imaging.

The treatment algorithm of the revised Fukuoka guidelines published in 2017 recommended that surgery should be considered in patients with high‐risk stigmata (HRS) or with worrisome features (WF) on imaging findings, such as the presence of a definitive nodule larger than 5 mm, MPD involvement on EUS findings, or cytology findings of suspicious or positive for malignancy.^12^ EUS is an imaging technique with high spatial resolution, and it is also capable of cytology, histology, and cyst fluid analysis via EUS‐FNA. However, its role in diagnostic algorithms based on revised Fukuoka guidelines was limited because EUS is not a procedure that can be performed at all institutions with the same quality. In recent years, the usefulness of EUS‐based imaging and EUS‐FNA in the diagnosis of IPMN has been widely reported. The Kyoto Guidelines published in 2023 proposed a more prominent role of EUS diagnosis in the management of IPMN and recommended its use in the evaluation of HRS and worrisome features^2^ (Figure 1).

### The utility of EUS in distinguishing malignant from benign IPMN compared to other diagnostic modalities

Contrast‐enhanced CT (CE‐CT; multidetector computed tomography [MDCT]) and MRI (contrast‐enhanced MRI/magnetic resonance cholangiopancreatography) have been considered the gold standard imaging methods for diagnosing the morphological features of IPMN.^12^ The most important finding in diagnosing high‐risk IPMNs is the presence of mural nodule (MN) or solid component (SC). MN is generally defined as a distinctly demarcated, elevated lesion within a cyst with contrast (existing blood flow) within the lesion on CT, MRI, or EUS, on the other hand, SC is defined as a substantial mass adjacent to the cyst wall and surrounding pancreatic parenchyma, and its finding suggests the presence of an invasive tumor, however, it is difficult to clearly differentiate between MN and SC.^2^, ^3^, ^13^ Kobayashi et al. reported that MN size measured by EUS is useful in differentiating benign from malignant IPMNs. In this report, they found significant differences in MN height (4.3 mm for benign vs. 16.4 mm for malignant, *p* < 0.0001) and width (5.7 mm for benign vs. 23.2 mm for malignant, *p* < 0.001) of the papillary growth.^14^ Recent reports indicate that EUS (including contrast‐enhanced EUS [CE‐EUS]) is comparable or superior to MDCT and MRI in the diagnosis of high‐grade dysplasia (HGD) and invasive carcinoma (IC) in IPMN.^15^, ^16^, ^17^, ^18^, ^19^, ^20^, ^21^, ^22^, ^23^, ^24^ (Table 1) Several studies have been reported comparing the diagnostic performance of imaging modalities in the diagnosis of MN/SC in IPMN. For example, Huynh et al. reported that MN diameter may be slightly overestimated by CT and MRI tomography and underestimated by EUS.^25^ Iwaya et al. reported that septal wall thickness observed by EUS correlates with IPMN grade and that the area under the curve (AUC) for differential diagnosis between benign and malignant IPMN was 0.70 for EUS and 0.56 for MDCT, indicating that EUS is superior to MDCT.^17^ Shimizu et al. considered EUS essential in the preoperative examination of IPMN because of its high spatial resolution, especially useful for the diagnosis of MPD dilatation and the presence of tiny papillary projections on the cyst wall or on the MPD.^26^ They reported a simple nomogram for predicting HGD/IC based on three factors: MN diameter, cyst diameter, and MPD diameter. In this study, they employed MN/SC size measured using EUS and reported that the malignancy probability of IPMNs with findings of MN 5–10 mm is less than 19%, while IPMNs with MN 10 mm or more have a malignancy probability increased to 67% by the MN factor findings alone.^26^ Kin et al. compared preoperative CE‐CT and EUS MN detection rates using pathological findings as the gold standard.^24^ This Japanese multicenter study enrolled 240 patients with pathological nodules. They could detect 83% of the MNs by EUS and only 53% by CT, concluding that EUS was significantly superior in detecting MNs. The average size of pathological MNs diagnosed by CT and EUS was 8 mm (range 1–50 mm) for CT, 8 mm (range 2–50 mm) for EUS, and 8 mm (range 3–50 mm) for CT. range 1–50 mm) and 6 mm (range 1–50 mm) by EUS. These results suggest that EUS is a highly sensitive diagnostic tool that allows visualization of smaller MNs.

**TABLE 1:** Comparison of diagnostic performance of imaging modalities in the diagnosis of intraductal papillary mucinous neoplasms.

Author	Journal	Year	Comparison item	Result
Nakagawa	Pancreas	2009	Tumor detection	MD‐CT = EUS > SD‐CT
Harima	World J Gastroenterol	2015	Mural nodule	EUS > CT = MRI
Kim	Pancreatology	2015	Mural nodule	EUS = CT
Du	World J Gastroenterol	2017	Detailed structure	EUS > CT, MRI
Ugbarugba	Pancreas	2018	Malignant potential	EUS > CT = MRI
Hwang	Eur Radiol	2018	Mural nodule	MRI > EUS
Iwaya	Dig Endosc	2019	Thickness of septum	EUS > CT
Ohno	Pancreatology	2019	MPD involvement	EUS > CT
Kin	Pancreatology	2023	Mural nodule	EUS > CT

Abbreviations: CT, computed tomography; EUS, endoscopic ultrasonography; MPD, main pancreatic duct; MRI, magnetic resonance imaging.

### The advantages of CE‐EUS/contrast‐enhanced harmonic EUS in IPMN diagnostics

**FIGURE 2:**
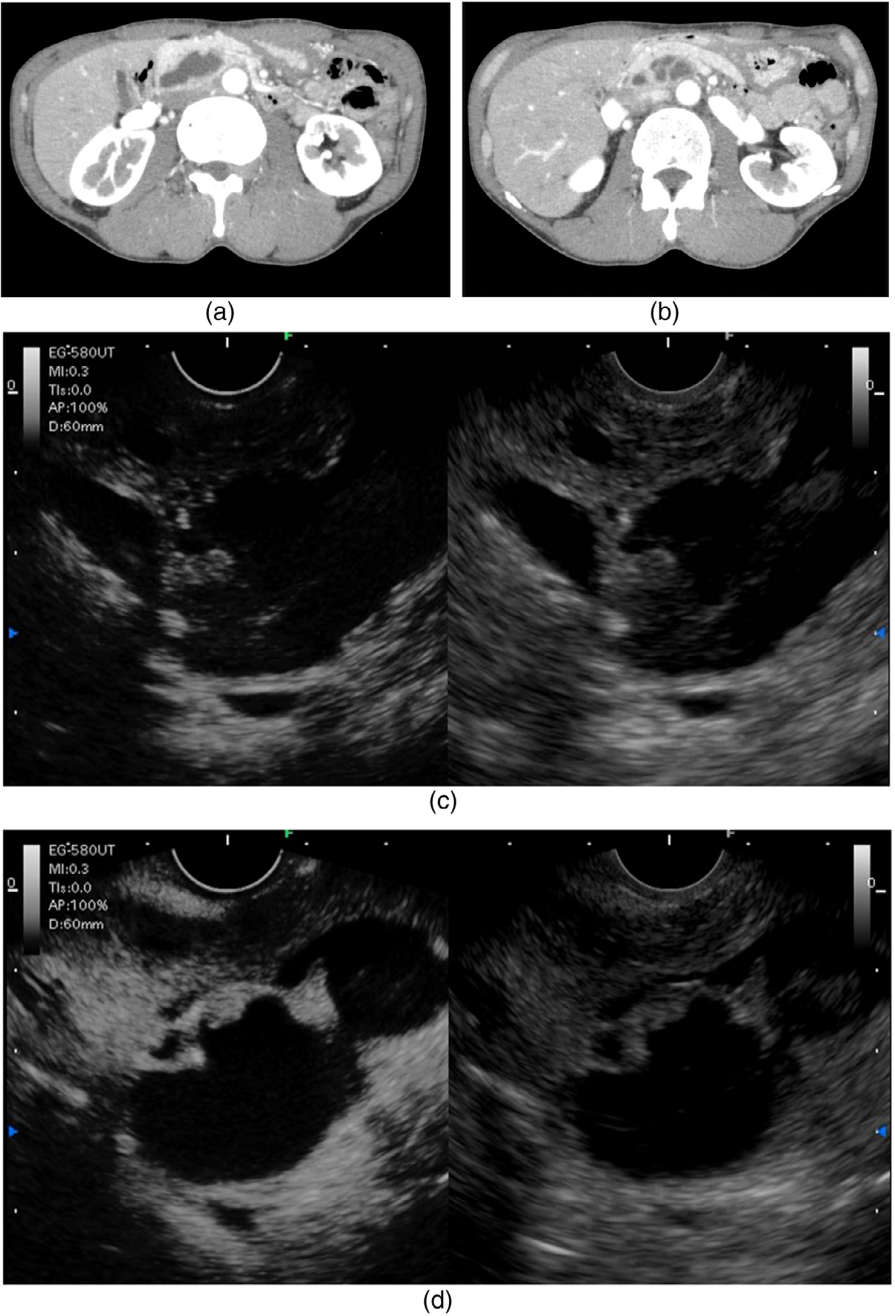
Contrast‐enhanced harmonic endoscopic ultrasonography (CH‐EUS) and contrast‐enhanced computed tomography (CE‐CT) for the diagnosis of mural nodules in intraductal papillary mucinous neoplasm (IPMN). (a) CT findings of mixed IPMN of the pancreatic head. The dilated main pancreatic duct and multilocular cystic lesions adjacent to the main pancreatic duct are seen. (b) CT findings of mixed IPMN of the pancreatic head. Septal structures are observed within multilocular cystic lesions. (c) CH‐EUS (before injection of Sonazoid). Right: reference B‐mode image, Left: contrast‐enhanced harmonic mode. The B‐mode and pre‐contrast harmonic EUS images show a hyperechoic polypoid lesion within the cyst. (d) CH‐EUS (after injection). Right: Reference B‐mode image; Left: Harmonic mode with contrast In CH‐EUS, the contrast effect is seen in the septal wall within the cyst and in the mural lesion, allowing the precise diagnosis of a mural nodule.

IPMN is a mucous‐producing tumor, and the mucous clots present in the cyst may be visualized as a polypoid mass. Distinguishing between a mucus clot and a true neoplastic nodule is essential for the assessment of the malignant potential of IPMN^15^ (Figure 2). “Enhancing MNs” is included in the HRS/worrisome features factor of the Kyoto guidelines.^2^ Historically, the diagnosis of enhancing MNs has been based on CE‐CT and contrast‐enhanced MRI findings. Recently, the use of CE‐EUS and contrast‐enhanced harmonic EUS (CH‐EUS) has been reported to differentiate between MN and mucus clots in PCLs^16^, ^20^, ^27^, ^28^, ^29^, ^30^, ^31^, ^32^, ^33^, ^34^, ^35^ (Table 2). There are two viewing methods for CE‐EUS examinations.^29^, ^36^, ^37^, ^38^ One uses the “color Doppler mode” to observe the enhanced blood flow signal (CE‐EUS). This method can detect blood flow information in the region of interest with high sensitivity, but quantitative evaluation of blood flow information is not possible. The other method is the “contrast‐enhanced harmonic method”, and observes the increase in echo brightness due to the inflow of contrast agent (CH‐EUS). Time‐intensity curve analysis can be used for quantitative evaluation in CH‐EUS.

**TABLE 2:** Diagnostic value of contrast‐enhanced endoscopic ultrasonography for intraductal papillary mucinous neoplasms (IPMNs).

Author	*N*	Contrast agent	Results
Hirooka	37	Albunex	Pattern diagnosis of PCLs, enhancement(+): SCN 3/3, mucinous cyst 6/8, and pseudocyst 0/5.
Hocke	125	SonoVue	Enhancement rate: Pseudocyst 6% (4/69) and cystic neoplasm 100% (56/56)
Fusaroli	76	SonoVue	Hyperenhancement: Serous cyst (86%) and mucinous cyst (89%) Hypoenhancement: Pseudocyst (90%)
Kamata	70	Sonazoid	Differential diagnosis of serous and mucinous cyst: Fundamental EUS = CH‐EUS
Yamashita	70	Sonazoid	Diagnosis of MNs: CH‐EUS sensitivity 100%, specificity 80%, and accuracy 94%
Harima	30	Sonazoid	Detection of MNs: CH‐EUS 98% (MDCT 92% and fundamental EUS 72%)
Yamamoto	30	Sonazoid	Malignant IPMNs by TIC analysis: higher echo intensity change, echo intensity reduction rate, and contrast ratio of the nodule/parenchyma.
Ohno	87	Levovist/Sonazoid	Classification of MNs by CE‐EUS, type III or IV correlated malignancy
Fujita	21	Sonazoid	Use for differentiating between MNs and mucous lumps
Ohno	166	Sonazoid	MPD involvement was diagnosed by CH‐EUS with a sensitivity of 83.5%, specificity of 87.0%, and accuracy of 84.9%. The accuracy of MPD involvement by EUS was superior to that of MPD size (84.9% vs. 71.7%).
Yamashita	115	Sonazoid	Detection of mural lesion: CH‐EUS 92% and CT 72%; Enhancement pattern is useful for the diagnosis of malignant IPMNs.
Yashika	183	Sonazoid	Multiphase evaluation using CEH‐EUS is a useful method for differentiating between invasive IPMC and PDAC.

Abbreviations: CE‐EUS, contrast‐enhanced endoscopic ultrasonography; CH‐EUS, contrast‐enhanced harmonic endoscopic ultrasonography; IPMN, intraductal papillary mucinous neoplasm; MNs, mural nodules; MPD, main pancreatic duct; PCLs, pancreatic cystic lesions; PDAC, pancreatic ductal adenocarcinoma.

Ohno et al. reported the morphologic classification of MNs based on CE‐EUS findings to exclude mucous clots. This study included 87 resected IPMNs. Type III (papillary nodule) and type IV (invasive nodule) MNs were associated with malignant IPMN in 88.9% and 91.7%, respectively. Multivariable logistic regression analysis showed that type III/IV MNs (odds ratio = 10.8; 95% confidence interval [CI] 2.75–56.1) and symptomatic IPMNs (odds ratio = 4.31; 95% CI 1.37–14.7) were significant for malignancy.^15^ Harima et al. evaluated the role of CH‐EUS in the diagnosis of branch duct‐type IPMN (BD‐IPMN) in 50 resected cases. CE‐EUS was performed when mural lesions were detected on EUS. By EUS observation, 28 of 50 IPMNs had mural lesions, and 15 cases were diagnosed with MNs by CH‐EUS. The accuracy of CT, EUS alone, and EUS combined with CH‐EUS for diagnosing MNs was 92%, 72%, and 98%, respectively. They reported that MN height measured by CH‐EUS (with a cut‐off value of 6.8 mm) improved the accuracy of differential diagnosis of benign versus malignant BD‐IPMN.^20^ Yamashita et al. compared the diagnostic performance of conventional EUS, CH‐EUS, and CE‐CT for the diagnosis of MN in IPMNs. CE‐CT had a sensitivity of 70%, specificity of 76%, and positive diagnosis rate of 83%, while CH‐EUS had a sensitivity of 97%, specificity of 76%, and positive diagnosis rate of 92%, indicating that CH‐EUS is superior in diagnosis.^34^ Additionally, it has been reported that CH‐EUS can observe and evaluate dynamic changes in contrast patterns in tumors and is useful for pathological grading of IPMN and differential diagnosis between IPMN‐derived cancer and pancreatic ductal adenocarcinoma (PDAC) by evaluating contrast patterns in MN/SC.^16^, ^17^ Yamamoto et al. investigated CH‐EUS with time‐intensity curve analysis for MNs in 30 cases of resected IPMNs and suggested malignant IPMNs exhibited significantly higher echo intensity change, echo intensity reduction rate, and nodule/parenchyma contrast ratio.^39^ Yashika et al. compared the contrast enhancement findings in 42 invasive IPMC cases and 141 PDAC cases through multiphasic analysis. After propensity score matching for stage and solid tumor diameter, contrast enhancement patterns were significantly more persistent in invasive IPMC than in PDAC (*p* = 0.0013).^35^

The malignancy rate of IPMN with MPD extension or involvement has been reported to be high. Marchegianni et al. used reviewed MPD involving positive IPMN based on pathologic findings and compared with histopathologic findings. IPMNs involving MPD had the predominant intestinal epithelial phenotype and 72% of them were malignant.^40^ In contrast to preoperative imaging, MPD involvement was defined by the diameter of the dilated MPD on preoperative imaging, mainly using CT or MRI. However, Crippa et al. reported that assuming MPD involvement in IPMN solely based on observing MPD dilation by CT or MRI analysis carries the risk of misdiagnosis, as they found that 26% of IPMN cases with an MPD diameter of over 5 mm were not concordant with pathological MPD involvement. Therefore, they recommended careful follow‐up of patients with MPD 5–9 mm because MPD dilatation in IPMN may be due to obstructive pancreatitis or secondary dilatation by mucinous masses.^41^ Ohno et al. classified MPD involvement into two definitions, one based on EUS findings of a low papillary lesion identified by CH‐EUS on MPD, and the other based on MPD size, and compared these definitions with pathologic findings. They concluded that MPD involvement based on EUS findings more accurately reflects pathological findings and can be a useful predictor of malignant IPMN.^16^

### Pathological diagnosis by EUS‐FNA and differential diagnosis of pancreatic cystic tumor by cystic fluid analysis

EUS‐FNA has been widely used in the pancreaticobiliary field, not only to obtain tissue for pathological diagnosis but also as an essential test for the practice of genomic medicine. EUS‐FNA for PCLs, especially IPMN, is recognized and practiced worldwide as a safe examination technique. The morbidity and mortality rates of EUS‐FNA for PCLs were 2.66% (95% CI 1.84–3.62) and 0.19% (95% CI 0.09–0.32), respectively.^42^, ^43^ In a prospective study of patients with pancreatic IPMN, Yoon et al. compared the frequency of peritoneal seeding with and without preoperative EUS‐FNA in patients undergoing IPMN resection. The results showed no difference in the frequency of peritoneal dissemination between patients who underwent EUS‐FNA and those who did not and concluded that EUS‐FNA is a safe technique in the diagnosis of pancreatic cystic tumors.^43^ However, there have been reports of needle tract seeding due to EUS‐FNA for cystic tumors, especially high‐grade lesions with MNs. Hirooka et al. and Yamabe et al. reported a case of needle tract seeding in patients with IPMC who underwent EUS‐FNA.^44^, ^45^ The Kyoto Guidelines point out the risk of peritoneal dissemination with EUS‐FNA. If the imaging findings by MDCT/MRI clearly show HRS findings and surgery is considered to be indicated, EUS‐FNA should not be performed.^2^

Regarding the diagnostic performance of cytological diagnosis using cystic fluid obtained by EUS‐FNA, a meta‐analysis showed that the sensitivity and specificity were 51% (95% CI 45%–58%) and 94% (95% CI 92%–96%), respectively (95% CI 92%–96%).^46^ Tanaka et al. reported a sensitivity of 57%, specificity of 84%, and AUC of 0.82 for EUS‐FNA in the diagnosis of HGD/IC as a result of a systematic review.^47^ De Jong K et al. reported that only one‐third of patients could obtain a sufficient sample volume for cytodiagnosis with EUS‐FNA. The low number of tumor cells in the cystic fluid is thought to be a contributing factor, and various tissue sampling methods have been reported to overcome this drawback.^48^ Recently, EUS‐guided through‐the‐needle biopsy (EUS‐TTNB) has been reported, in which biopsy forceps are inserted through the lumen of the EUS‐FNA needle to perform cyst wall biopsy. Tacelli et al. reported a positive diagnosis rate of 86.7% for EUS‐TTNB in pancreatic cystic tumors, but an incidental complication was observed in 8.6% of cases.^49^ Shi‐yu et al. compared the diagnostic performance of EUS‐related procedures for pancreatic cystic tumors using a network meta‐analysis approach.^50^ The results showed that EUS‐TTNB had a sensitivity of 0.87 and specificity of 0.83 for the diagnosis of mucinous PCL, and a sensitivity of 0.97 and specificity of 0.95 for the diagnosis of malignant PCL. On the other hand, EUS‐TTNB is associated with incidental findings such as intracystic hemorrhage and pancreatitis, which occurred in 0%–22.9% of cases.

The analysis of cystic fluid for molecular markers and gene profiling including pancreatic enzymes and carcinoembryonic antigen (CEA) in cystic fluid has been found useful in the differential diagnosis of PCLs.^51^, ^52^, ^53^, ^54^ The CEA level in a cystic fluid is higher in mucinous cysts, and in the differential diagnosis of IPMN between benign and malignant tumors, the sensitivity and specificity were reported to be 90% and 71%, respectively, when CEA >200 ng/mL was used as the cutoff and 87.5% sensitivity and 73% specificity when CA72‐4 >40 U/mL was used as the cutoff. However, the cutoff values for tumor marker levels in pancreatic cysts vary among reports.

In recent years, the usefulness of gene profiling in cystic fluid as a liquid biopsy using cystic fluid has also been reported. Detection of *KRAS* and *GNAS* mutations in cystic fluid has been reported to have a sensitivity of 79% and specificity of 98% for the diagnosis of mucinous cysts as a useful tool in the differential diagnosis of PCLs.^55^, ^56^, ^57^, ^58^ In the diagnosis of high‐grade IPMN (HGD/IC), evaluation of *TP53, SMAD4, CDKN2A*, and *PIK3CA* mutations, which are also relatively common in PDAC, is useful in identifying the presence of HGD/IC with low sensitivity (3%–39%) but high specificity (92%–98%).^54^

In summary, EUS‐FNA is useful for analyzing pancreatic cystic tumors, but because of some risk of needle tract seeding, the technique should only be performed if the test results are useful in determining the patient's treatment strategy.

## FUTURE PERSPECTIVES

EUS is a diagnostic imaging method with high spatial resolution, but it may have a weakness in that the obtained image findings and diagnostic imaging decisions depend on the surgeon and lack objectivity. Machine learning using artificial intelligence, which has been advancing rapidly in recent years, maybe a solution to this problem. For the differential diagnosis between benign and malignant IPMN using EUS images, the usefulness of deep learning for overcoming poor objectivity has already been reported.^59^, ^60^ Schulz et al. performed machine learning using 3355 EUS images from 43 patients. The diagnostic performance of AI for high‐grade IPMN/Invasive carcinoma was reported to be 100% sensitivity, 99.7% specificity, and 99.6% positive predictive value, which is significantly superior to the predictive performance of various IPMN guidelines.^60^ However, even with advances in AI diagnostic technology, it is essential for endoscopists to have knowledge of appropriate EUS maneuvers and ultrasound imaging in order to use the full potential of EUS imaging and EUS‐FNA procedures.

## CONFLICT OF INTEREST STATEMENT

None.

## ETHICS STATEMENT

As this article is a review article based on published literature, no ethics approval was required.

‐Approval of the research protocol by an Institutional Reviewer Board. N/A

‐Informed Consent. N/A

‐Registry and the Registration No. of the study/trial. N/A

‐Animal Studies. N/A
